# Medical Students’ and Physicians’ Attitudes toward Patients’ Consent to Participate in Clinical Training

**Published:** 2015-01

**Authors:** ATHAR OMID, PARNAZ DANESHPAJOUHNEJAD, OMID PIRHAJI

**Affiliations:** 1 Department of Medical Education, Medical Education Research Center, Isfahan University of medical sciences, Isfahan, Iran; 2 Isfahan Medical Students’ Research Center, Isfahan University of Medical Sciences, Isfahan, Iran

**Keywords:** Medical students, Physician-patient relations, Informed consent, Attitude

## Abstract

**Introduction:** The responsibility of the medical training team towards a patient referring to an academic medical center has not been fully clarified. In this article we have looked at current practice in Medical University of Isfahan and evaluated the attitude of the medical team towards patients’consent to be involved in medical students’education.

**Methods:** In this cross-sectional study, conducted in 2012, we distributed self-administrated questionnaires among medical mentors, residents and students of academic hospitals in Isfahan, Iran. This researcher-made questionnaire consisted of several questions concerning dimensions of informed consent. The data were analyzed, using independent t-tests and ANOVA.

**Results: **Ninety-one medical students (51 females) and 61 members of medical training team (25 females) completed the questionnaires. The overall average attitude score was 36.53±5.89 out of 60, which is classified as fair. The average attitude score for medical students and mentors were not significantly different. The average attitude score of the female students was classified as good, and was different from that of male students, significantly (p<0.05). By categorizing subjects into those with≥5 years of managerial or educational experience and those below 5, a significant difference in average attitude score was documented (35.8±2.54 in≥5and 34.0±2.9 in>5group).

**Conclusion:** The attitude of the medical team is thoroughly far from what is expected. Thus, the need to provide both medical students and medical mentors with data on the importance of obtaining patients’ consent to be involved in medical education is highlighted.

## Introduction

Patient-based teaching is a way of educating medical students to learn clinical skills in the presence of real patients, thereby students learn all the three scopes needed to manage a doctor-patient relationship, i.e. knowledge, skill and attitude. They will also be taught how to act in their future day-to-day performances (1, 2). As the learning is established within context on real patients, students learn many interpersonal skills and what they learn will be therefore easier to recall (1).

In the beginning of medical education, involvement of medical students in patient care includes mere observation and shadowing but later on, they would take on more responsibilities such as monitoring the patients’ condition and even being directly involved in the treatment of a patient so that the students’ limited skills are counter-balanced by supervision of the medical team (2-4).

Patients’ participation in medical education, including lectures, tutorials, student rounds and consultations, does not involve ethical controversy in most occasions but at times, the patient involvement may get complicated and problematic (5). In some medical centers, we may see patients, even clinically unstable ones, being supervised by individuals with no clinical training, and this might affect the patients adversely (2, 6-8). Furthermore, it is inappropriate to assume that all the patients would choose to actively participate in the medical students’ education, just because they were admitted to an academic medical center. Nowadays primary care has been expanded so much and it can be considered as a setting for training doctors. Yet some patients may feel aggrieved that students are involved in their treatment (3, 4, 9).

Having this knowledge, and bearing in mind that the extensive patient contact needed for medical education cannot be achieved only by opportunistic patient contact (4), the importance of informed consent and confidentiality in all aspects of patient care is clarified (2, 10). The elements of consent include understanding the procedures as well as the risks and benefits of these procedures (11). It is also worth mentioning that by respecting patients’ autonomy, and being responsible towards patients’ rights, the patients would be more willing to take part in medical students’ teaching process, and receive an adequate and standard level of care delivered by competent staff (7).

Having insufficient respect for patients’ autonomy is problematic (5). It is assumed that this climate demands direct attention of both medical mentors and medical students. To our knowledge, the responsibility of the medical training team and practice staff towards a patient referring to an academic medical center has not been fully clarified (12). Moreover, the attitude towards patients may vary among different institutions. Thus, in this article we have looked at the current practice in Isfahan University of Medical Sciences and tried to elaborate and compare the attitude of medical teachers and students towards patients’ consent to be involved in the education of medical students.

## Methods

In this cross-sectional study, conducted in February-March 2012, we distributed a self-administrated questionnaire among 100 randomly selected medical mentors and residents of academic hospitals of Isfahan University of Medical Sciences. Another self-administered questionnaire evaluating the same aspects, but with minimal changes to be appropriate for the target population, was distributed among 100 randomly selected 4th to 7th year medical students, who were interns and students receiving clinical training. The data-gathering tool was a researcher-made questionnaire developed based on literature review. Search keywords to refine results were (clinical) AND (patient right OR confidentiality OR choice OR privacy OR consent) AND (Teach* or Educat*) and electronic databases including PubMed, Scopus, Wiley, Proquest, EMBASE and ISI Web of Knowledge were searched. We also looked for evidence in books on this field and the final questionnaire was then developed.

Both questionnaires comprised two parts of demographic factors and questions related to patients’ rights. The demographic factors assessed in the study were sex, major, years of managerial or educational experience, and academic rank for medical mentors and age, sex, marital status, education level, and grade point average (GPA) for students. The latter part of the questionnaires (Table 1) consisted of twelve 5-item questions covering several dimensions of informed consent (13), including choice (3 questions), information (5 questions), understanding (2questions), and continuous (2 questions). The 5-item questions (strongly disagree=1, disagree=2, neither agree nor disagree=3, agree=4, strongly agree=5) led to a maximum attitude score of 60. The total attitude scores were recoded into four categories: poor (12-24), fair (25-36), good (37-48), and excellent (49-60).

The validity of the questionnaires was approved by a panel of six experts in the field and the Cronbach's alpha reliability coefficient was 0.78. All the questions, except two had negative connotation. To calculate the average attitude score, the answers to each of these two questions were recoded so that higher scores denoted less agreement with the phrase, i.e. the values of each variable would range in a descending order from strongly disagree to strongly agree. The data gathering process was confidential and anonymous.

The data were analyzed using SPSS version 14. Continuous and qualitative variables were expressed as mean±standard and proportions (%), respectively. The association between demographic factors and the questionnaire scores was analyzed using independent t-tests and ANOVA. A two-sided α level of 0.05 was used to assess statistical significance.

**Table 1:** The questionnaire administered to the subjects, comprising three dimensions and 15 questions

**Dimension**	**#**	**Question**
Choice	1	Professors should confront the patients who do not allow medical students to examine them.
	2	The patient should not be concerned about gender conformity with medical students examining them.
	3*	The patient has the right to accept or reject examination by the medical student.
Information	4	By accepting to be examined by the medical student, the patient has implicitly accepted the possibility of physical injury.
	5	By accepting to be examined by the medical student, the patient has implicitly accepted the possibility of emotional injury.
	6	Medical students should not tell the patients that they are examining them for the purpose of learning, because patients would not let them do that.
	7	Before examination, the patient need not be aware of medical students’ experience level.
	8*	The patient should be aware of being examined by medical students, not the real doctor.
Understanding	9	A patient accepting to be admitted to an educational center indirectly has agreed to be examined by medical students.
	10	Asking for permission to examine the patient will disrupt the learning process.
Continuous	11	Examining the patients who cannot or do not have the possibility to cancel the examination is a good opportunity to improve the medical students’ skills.
	12	Only the patient has the right to agree or disagree with the examination process, not his or her accompanying person.

* Recoded questions

## Results

Ninety one medical students (51 females and 40 males) (response rate=91%) with a mean age of 23.48±0.99 and sixty one members of the medical training team (25 females and 35 males) (response rate=61%) with 7.33±5.77 years of managerial or educational experience, completed the questionnaires. The demographic factors and mean ± standard deviation of average attitude scores in different groups of study are displayed in Table 2. The overall average attitude score was 36.53±5.89 out of 60, which is classified as fair. The average attitude scores for medical students and mentors were 37.12±6.57 and 36.06±3.50, respectively, but they were not significantly different.

Interns, with the average attitude score of 39.90±2.92, had a statistically significant difference (p<0.005) with other medical students (4^th^ and 5^th^ year) with an average attitude score of 35.52±7.64. Moreover, the average attitude score of female students was 38.33±5.38, which was classified as good, and was significantly different from that of male students (p<0.050). But there was no significant difference in the average attitude score of married versus single students. Students with GPA above and below 17 were not significantly different in terms of average attitude score, either. The average attitude score for subjects with GPA below 17 was 36.69±6.87, and 37.79±4.56 for subjects with GPA above 17 (p=0.500).

The difference between average attitude scores of professors, associate professors, and assistant professors was not statistically significant. By categorizing subjects into those with ≥5 years of managerial or educational experience and those below 5, a significant difference in average attitude score was documented (35.8±2.54 in ≥5 and 34.0±2.9 in >5 group). There was no significant difference between mentors of internal medicine (34.74±2.99) and surgery (35.25±2.25) in terms of average attitude score (p=0.600).

In Table 3, the distribution, mean±standard deviation of attitude scores for each question is reported separately.

Among medical mentors the lowest attitude scores were assigned to questions 1,2,6,7,8,11, regarding the dimensions of choice, information, and continuous, with 2.29±1.13, 2.21±0.79, 2.03±0.74, 2.16±0.83, 1.8±0.63 and 2.15±0.88 scores respectively.

Medical students got the lowest scores for questions 2, 3, 7, 8, 9, about choice, information, and understanding dimensions, with 2.05±0.90, 2.36±1.04, 2.01±0.71, 2.51±1.04, 2.73±1.05 scores, respectively.

**Table 2:** The average attitude score in each group of subjects (mentors and medical students) based on the demographic factors gathered

		Sex		Education level		Marital status		Total
		Female	Male	4th and 5th year students/ Residents	Interns/ Professors	Married	Single	
Students	Percent	55.8%	44.2%	64%	36%	88.5%	11.5%	100%
	ATS	38.33±5.38	35.26±7.86	35.52±7.64	39.90±2.92			37.12 ± 6.57
Mentors	Percent	25 (41%)	36 (59%)	26 (42.5%)	35 (57.5%)			100%
	ATS	35.55±2.74	34.54±3.6	35.58±3.35	33.88±3.03			36.06± 3.50

ATS=Average attitude score

The difference in different sex groups and education level groups in students was statistically significant.

**Table 3:** Answers of both groups of subjects to questions 1-15 and mean±SD

Dimension	Question	Agree or strongly agree (%)		Neither agree nor disagree (%)		Disagree or strongly disagree (%)		Mean±SD(1–5)	
		Mentors	Students	Mentors	Students	Mentors	Students	Mentors	Students
Choice	1	65.6	38.5	13.1	17.6	21.3	44.0	2.29±1.13	2.96±1.20
	2	59.0	74.8	39.3	17.6	1.6	7.7	2.21±0.79	2.05±0.90
	3*	55.0	18.9	28.3	14.4	16.6	66.7	3.66±1.25	2.36±1.04
Information	4	6.5	7.8	19.4	12.2	66.2	80.0	3.87±0.84	3.79±0.68
	5	0.0	12.5	21.3	19.3	78.7	68.2	4.14±0.67	3.46±0.80
	6	73.8	40.0	24.6	16.7	1.6	42.4	2.03±0.74	3.00±1.16
	7	67.2	80.9	26.2	15.7	6.6	3.4	2.16±0.83	2.01±0.71
	8*	0.0	23.9	11.7	13.6	88.4	62.5	1.80±0.63	2.51±1.04
Understanding	9	37.7	50.0	27.9	20.0	34.4	30.0	2.93±1.07	2.73±1.05
	10	8.1	20.9	24.2	9.9	64.5	69.3	3.80±0.93	3.67±1.16
Continuous	11	58.0	34.1	37.7	24.2	3.2	41.8	2.15±0.88	3.03±1.08
	12	6.5	31.1	19.7	25.6	80.0	43.3	3.98±0.81	3.08±1.02

*Recoded questions

## Discussion

Most of the studies previously performed on patients’ consent to get involved in medical education were conducted on the patients themselves, and little data exists on the medical team (trainers and trainees) attitude toward patients’ consent. In this study we aimed to move beyond the existing data and tried to evaluate the attitude of medical team towards patients’ consent in the educational processes.

According to this study, the overall average attitude score was classified as fair, which shows that although medical associations insist on the importance of patients’ consent to being involved in medical education while receiving medical care (11), and surveys of patients have shown that patients feel it is important to know that a medical student is participating in their medical care (14), the medical team may not inform patients of such procedures and medical students hesitate to disclose their identity because they assume that patients may not allow them to take part in their medical care. Another reason may be the strong belief among the medical team that patients admitted to educational hospitals pay less money than those admitted to private hospitals, and this makes it logical to be involved in the education of medical students or even different procedures performed by a medical student who has never performed such a procedure before. But studies show that while a medical student may have the right to clinical education, the obligation to fulfill this right rests on the medical university and not on the patients of its teaching hospitals (15, 16).

In our study, the average attitude score of medical mentors was lower than that of the medical students, which shows that medical mentors believe that the students’ right to learn is more important than the patients’ right to be informed about the skills of those involved in their care. However, interns had a higher average attitude score than 4th and 5th year medical students, which proves that as medical students get more involved in patients’ care, their sense of responsibility to inform patients that they are students is increased. This may be because, as reported in previous studies (11), majority of patients may consent to simple procedures by inexperienced medical students. Thus, medical students feel more comfortable to reveal their identity to the patients. Moreover, most of the medical students may not know how to introduce themselves to the patient, to create a sense of confidence and start a good doctor-patient relationship. This emphasizes the importance of providing medical students with a guideline on how to introduce themselves to patients. It should be emphasized that as it is included in patients’ rights to know the clinical skills and level of knowledge of those involved in their medical care, depriving the patients of having such data is not ethically accepted.

The other point that has to be discussed is that both medical students and mentors held the belief that patients should not be concerned about gender conformity with the medical care providers. But studies have shown that one of the major concerns of patients and reasons to avoid participating in medical education of students, is that they prefer to be examined by the same sex (17, 18). Thus, it is necessary to inform the patient about the gender of the student who is going to examine them.

In this study we had several limitations. This study was carried out in one hospital and only a small number of hospital workers participated in the study. Further multicentric studies with larger populations are needed to confirm the results. 

## Conclusion

According to this study, the attitude of the medical team is thoroughly far from what is expected. Thus, the need to provide both medical students and medical mentors with data on the importance of obtaining patients’ consent to be involved in medical education is highlighted. The medical team should know that informing the patients not only does not interfere with medical education, but also helps improve the patients’ behavior towards students, because they may feel pleased by taking part in the education, which provides the society with future doctors. 
